# COVID‐19, the Great Recession and Economic Recovery: A Tale of Two Crises

**DOI:** 10.1111/jcms.13383

**Published:** 2022-07-07

**Authors:** Hilary Ingham

**Affiliations:** ^1^ Department of Economics Lancaster University Lancaster

**Keywords:** COVID‐19, the Great Recession, labour market support, flexible working

## Abstract

COVID‐19 caused a major economic downturn, the like of which had not been seen since the Great Recession although the underlying causes of the two crises were very different; systemic risk versus a virus. Here we look at how flexible work practices, allied with adequate supports and lifelong learning opportunities, aided economic recovery following the earlier crisis in order to see if there are any lessons to be learnt for post‐pandemic recovery. Overall, the results indicated that flexicurity provided a modest growth dividend during the Great Recession, typically no more than one percentage point. Of the individual components, the short‐run results indicated that security along with life‐long learning and part‐time work proved the most beneficial, although flexible work practices also boosted growth, albeit to a lesser extent. For flexible labour markets, the long‐run results indicated that the growth gains were highest in trusting economies with, or without, social partner engagement.

## Introduction

More than two years have passed since the first cases of COVID‐19 were reported. Despite an initial strong worldwide recovery, rising energy prices, the war in Ukraine and ongoing supply shortages threaten future growth. One means by which to ameliorate this is through increasing productivity and it has been suggested that countries look to Denmark, a country historically known for flexicurity, as a model to manage post‐pandemic labour market change (Brookings, 2020). This paper looks back to the last major worldwide economic crisis, the Great Recession, and examines the success, or otherwise, of the measures European nations adopted to ameliorate the labour market imbalances brought about by the financial crisis. In particular, we look at whether flexibility and security – flexicurity – aided economic recovery as well as examining the role of the social partners in facilitating adjustment following the shock and the degree to which trust is an important factor promoting economic recovery.

Central to the organization of work is the relationship between employers and employees which may rupture when the labour market faces a severe shock, a situation exacerbated by the fact that labour contracts are implicit and incomplete. As Zeitoum and Pamini (2021) note, recessions can substantially increase the pressure on employers to breach implicit contracts since they are unwritten and unenforceable. Social partners are key players in any intermediation regarding changing work practices, although Lorenz (1999) stresses that there is no guarantee that cooperation will succeed, even when there are potential mutual gains, if there is an absence of trust.

The evidence presented in this paper shows that it is the security elements – labour market supports and lifelong learning – along with part‐time working that were the elements of flexicurity that promoted the most growth after the last recession. This result remained robust when social partner engagement and trust were added to the model. Similar findings emerged for flexible work practices although their impact was smaller and statistically weaker.

The paper begins by looking at the similarities, and differences, in the two recessions and presents some key statistics covering output and the labour market. Section I considers whether elements of flexicurity might aid post‐pandemic recovery and details the variables used in the empirical analysis. Section II considers the intermediation role of the social partners and questions whether their position has been weakened following the financial crash. The section also looks at whether a trusting environment may contribute to the success of a flexicurity policy agenda. The empirical model, along with the results, follow in the next section with Section IV looking at the pandemic experiences of the countries with high levels of flexicurity at the end of the Great Recession. A summary and conclusion ends the paper.

## I COVID‐19 and the Great Recession

The roots of the Great Recession lay in systemic risk which emanated from the US sub‐prime housing market. In 2008/9, EU GDP fell by 4.2 per cent (European Commission, 2010) although the aggregate figure conceals marked variation with Germany, Italy and the UK recording year‐on‐year falls in the range of 6–7 per cent whereas, in Poland, growth remained positive, albeit slowing. The Baltic States fared particularly badly with Estonia and Latvia suffering GDP declines of 15 per cent and 20 per cent respectively (European Commission, 2010). The labour market effects of the Great Recession also varied. Both Ireland and Spain experienced increases in their unemployment rates of around 7.5 percentage points. In contrast, Germany's unemployment rate fell as jobs were sustained by the country's short‐term working *Kurzabeit* programme, although a recent estimate (IMF, 2020) suggests that only one‐third of the reduction in working time was directly attributable to the scheme, with the remainder due to other margins of flexibililty. However, by 2010, around 23 million individuals in the EU were unemployed, which represented close to 10 per cent of the working age population (European Commission, 2010).

Unlike the Great Recession, COVID‐19, which first appeared at the end of 2019, was an exogeneous shock. In general, the pandemic caused sharper falls in output than did the Great Recession. The UK and Spain suffered negative growth of 19 per cent and 18 per cent respectively in the third quarter of 2020; the two countries with the highest death tolls in Europe. Conversely, Sweden, a country that resisted lockdown until January 2021, even grew in the first months of the pandemic. Furthermore, the bounce‐back during the pandemic period was strong on the back of pent‐up demand emanating from lockdown restrictions. The labour market impacts of the pandemic were very different from those experienced in the financial crisis. Despite the fact that employment and total hours worked declined at the sharpest rates on record, between February and October 2020, unemployment in the Euro area only increased by a modest 1.2 percentage points to 8.4 per cent (ECB, 2020).

To counteract recessions, governments have fiscal measures available which work in conjunction with automatic stabilizers. On the demand side, the immediate priority is to stimulate aggregate demand with the focus being on the overall economy as opposed to specific sectors, businesses or individuals. In the longer term, supply side policies can be employed to improve the economy's productive potential. Thus, the tax system can be used to incentivize factor outputs and measures can be put into place to reduce labour market rigidities and improve human capital. In terms of government responses to the Great Recession, the general belief was that there was a high need for fiscal stimulus (Guajardo *et al*., 2014) as not only was the resilience of national economies impaired by both the depth and global nature of the shock, agents faced limited access to, or a high cost of, self‐insurance through financial institutions and credit markets and the zero‐bound on nominal interest rates constrained monetary policy.

Although short‐time working schemes were adopted in Germany, Italy and the Netherlands during the Great Recession, early in the pandemic there was a wide‐scale roll‐out of job retention schemes wherein workers reduced their hours, sometimes to as low as zero, and received a proportion of their salary with firms often making up some, or all, of the difference. The one country that adopted a very different policy response was the US where employees were ‘let go’, but individuals were supported by cash payments. These schemes protect worker incomes and cushion aggregate demand. They allow firms to retain human capital and reduce the problem of costly separations, re‐hiring and training. However, there is a deadweight effect if a scheme is actually subsidizing a worker who would have retained their job anyway. There is also a potential displacement effect if workers are supported in jobs that have no long‐term viability.

Recovery following the Great Recession was slow. In 2009, value added in the EU‐27 fell by some 4 per cent and it was not until 2011 that it returned to its pre‐crisis level whereupon it stagnated until 2013 due to sluggish recovery in aggregate demand and the labour market. The fiscal response came in two parts (Bozio *et al*., 2015). First, countries adopted active fiscal stimuli packages aimed specifically at limiting the length and the depth of the recession. However, they also looked to more permanent fiscal consolidation to restore the sustainability of public finance positions. For the financial crisis, Coenen *et al*. (2012) actually found a negligible role for discretionary fiscal policies within the Eurozone. However, using an enriched version of the ECBs New Area‐Wide Model, which is calibrated with a high degree of detail, they estimated that such policies increased annualized quarterly real GDP growth by up to 1.6 percentage points during the crisis.

Although fiscal consolidation measures were viewed as expansionary by some (for example Alesina and Ardagna, 2010), Blanchard and Leigh (2014) provided contradictory evidence that both the IMF and the EU had underestimated the negative growth effects of austerity policies by about one percentage point. Using a multi‐country DGSE model, House *et al*. (2020) estimate that had ten of the EU economies (Belgium, Germany, Estonia, France, Luxembourg, Netherlands, Austria, Slovenia, Slovakia and Finland) not experienced austerity shocks, aggregate output in these countries in 2014 would have been roughly equal to its pre‐crisis level, as opposed to an output loss of 3 per cent. Furthermore, output losses in the GIIPS economies (Greece, Ireland, Italy, Portugal and Spain) would have been cut from almost 18 per cent below trend by the end of 2014 to only 1 per cent below trend.

In the following section attention turns to examining whether flexicurity, which embodies certain supply side fiscal measures, contributed to economic growth after the financial crash. In principal, flexicurity fosters innovation‐led growth by making creative destruction easier to implement and also more efficient due to the accompanying public investment in training. Furthermore, it also renders innovation‐driven growth more protective and inclusive by providing income support and training, facilitating laid‐off workers' re‐entry into employment.

## II Might Flexicurity Help the Post‐pandemic Labour Market?

Flexicurity covers aspects of both economic and social policies involving effective policy design covering flexible and reliable contractual arrangements; comprehensive lifelong learning strategies; effective active labour market policies and modern social security systems. The theoretical underpinnings of the relationship between labour flexibility and growth at the firm level are in Bentolila and Bertola (1990) and explored in a general equilibrium framework by Hopenhayn and Rogerson (1993). The argument is that highly regulated labour markets constrain the flexibility of a firm's employment policy making hiring a risky proposition due to the costs of terminating contracts. As such, labour market distortions can encourage firms to use labour inefficiently causing productivity to fall. Obviously, such distortions can improve workers' welfare by stabilizing employment, but this loss of flexibility comes with the cost of reduced efficiency. At the company level, flexicurity is, in principle, designed to simultaneously appeal to employers and employees alike. However, in practice there tends to be scepticism, with trade unions viewing the policy as a means to deregulate labour markets and erode worker protection. Conversely, employers view it as a means to manage volatile and uncertain demand.

There are four key components of flexicurity: flexible contractual arrangements, comprehensive lifelong learning strategies, effective policies to help the unemployed re‐enter the labour market and modern social protection systems. The flexible workforce comprises those employed on temporary contracts (TEMP), the self‐employed (SELF), those working part‐time (PART), or who are classified as unpaid family workers (UFW). Data for each country are available in the Supporting Information that accompanies this paper but the picture is one of large differences in the penetration of flexible contractual forms across EU members. Part‐time work is most prevalent in the Netherlands, with the average for the period exceeding 40 per cent, but less common in the central and eastern European (CEE) countries; only 2 per cent for Bulgaria. Spain recorded the highest incidence of temporary positions, with a figure of 24 per cent with Poland recording a figure of over 17 per cent. This country excepted, temporary work was rare in CEE countries. Self‐employment was highest in Greece, standing at almost 30 per cent, whereas the comparable figures for Denmark, Estonia, Luxembourg and Sweden were all less than one‐third of this. UFWs were universally low with the exception of Romania with a figure of almost 13 per cent.

LLL covers all learning activity undertaken throughout life, with the aim of improving knowledge, skills and competences within a personal, civic, social and/or employment‐related perspective. Some of the highest rates for lifelong learning are in the Nordic countries, standing at over 20 per cent in Denmark, Finland and Sweden. In general, CEE nations have a poor record in adult learning; only Slovenia has more than 10 per cent of its target population participating. However, Greece performs very poorly on this metric only managing to outperform the two 2007 entrants, Bulgaria and Romania.

Active Labour Market Policy (ALMP) expenditure comprises labour market policy interventions covering services to aid the ‘activation’ of the unemployed and the economically inactive and job maintenance for those threatened by unemployment. Training, job sharing and rotation, job creation and employment incentives are among the initiatives covered. Passive labour market spend (PLMP) captures income security and covers out‐of‐work income maintenance and support and early retirement. In excess of 90 per cent of these funds are channeled to cash payments to support the unemployed during active job‐search, primarily through unemployment benefit. Spending on ALMP is low across the Union and only exceeds €8,000 in PPS in Denmark. Rather more resources are targeted at PLMP with Belgium, Denmark and Luxembourg being the highest spenders with figures in excess of €9,000 in PPS. In general, such spending is low in the more recent entrants to the EU.

Little systematic work exists on the overall effect of flexicurity on economic performance. Michie and Sheehan (2003) found the use of flexible contracts and rates of labour turnover were associated with reduced levels of innovative activity, which might be expected to be detrimental to growth. On the security side, Dolenc and Laporšek (2013) found spending on ALMP to exert a positive influence on labour and total factor productivity. Focusing on OECD countries over the period 1993 to 2005, Kim *et al*. (2015) used K‐means clustering to group countries on equity and efficiency. Among the efficiency variables were fixed‐term and agency workers, union decentralization, ALMP expenditure and unemployment insurance. Growth, employment rates and labour productivity appear within the efficiency set. Their results refute the suggestion that greater efficiency comes at the expense of equity. Denmark, for example, consistently scored highly on both whereas Turkey and Italy scored poorly on both.

Using data from the 2009 European Company Survey, Wang and Heyes (2017) found that employment security and job continuity were associated with better labour productivity. The best performers used no fixed‐term workers but firms who did, but retained them at the end of their contracts, out‐performed companies who released them. Also, a good relationship between employee representative bodies and managers increased the propensity to retain those on fixed‐term contracts.

In terms of the efficacy of flexicurity as a panacea in times of recession, some of the countries classified as having flexible labour markets recorded the highest increase in unemployment rates during the financial crisis. Auer (2010) highlights the poor performance of Denmark in terms of GDP growth and employment. In actual fact, the Danish economy was already slowing down prior to the financial crash, amidst a correction in the housing market. Output fell by more than 7 per cent between 2008 and 2009 as exports and investment collapsed, private consumption contracted, unemployment rose and house prices fell (European Commission, 2013). It should though be noted that Jensen (2017) contends that flexicurity in Denmark had passed its peak by the time the concept entered the common lexicon.

In addition to the flexicurity components discussed above, two other factors merit consideration. First, the social partners who act as intermediaries between management and employees and who may be central to the success, or otherwise, of firms' efforts to introduce more flexible work practices. Second, is the degree of trust between the parties involved in various facets of the flexicurity construct.

## III The Role of the Social Partners and Trust

Labour organization is often mediated by employee representatives such as unions. However, contracts between a firm and its workers are incomplete and implicit and as Zeitoum and Pamini (2021) note, a severe recession represents an external shock increasing pressure on employers to renegade on any implicit bargains. Thus, social partners play a vital role in shaping working conditions through collective bargaining and tripartite social dialogue. During recessions, they will be involved in key negotiations on matters such as redundancies, wage reductions and short‐time working.

For the Great Recession, Glassner and Keune (2010) highlight four areas where they played key roles. First, the safeguarding of jobs via flexible reductions in working time. Second, by increasing employment through vocational training and re‐skilling. Third, by facilitating changes in work organization supporting company restructuring programmes and, fourth, by allowing temporary deviations from collectively agreed pay rules. Using data from the Workplace Employment Relations Survey (WERS), Zeitoun and Pamini (2019) examined the impact of union voice (consultation over pay, hours, holiday, pensions, training, health and safety) and HRM practices (participation, team working, employee development selection and incentives) on firm performance uncovering a positive relationship, but only during recessions.

It is argued that the Great Recession fractured industrial relations systems within Europe because austerity packages came with conditions imposed by the troika of the EC, the ECB and the IMF (Marginson, 2017). So, the Memoranda of Understanding covering debt relief to Greece, Portugal and Romania, included de‐centralization and abolition of the extension mechanism for collective bargaining (Marginson and Weiz, 2015). Likewise, the European Semester has noted aspects of collective bargaining practices in need of reform (Marginson and Weiz, 2015).

In order to capture the extent of social partner engagement, Eurofound's Industrial Democracy Index (ID) (Eurofound, 2018) is used. This encompasses four dimensions. First, the autonomy of the social partners in collective bargaining. Second, their representation rights at both the macro level (collective bargaining and social dialogue) and at company level (works councils etc.). Third is their participation in company level management decision‐making. Fourth is their ability to exert influence in the context of collective bargaining or management decision‐making. The index lies in the 0 to 100 range with higher values representing a greater degree of industrial democracy. Data is available for 2008–12 and 2013–17 and so backwards linear extrapolation is used for earlier years. There is little variation across the two periods with the largest being a three‐percentage point difference for Bulgaria. Denmark, Sweden and the Netherlands occupied the top three positions with values close to 70 per cent. Latvia, Romania and Bulgaria occupy the bottom positions with figures of around 40 per cent.

Of course, relationships between stakeholders in a firm do not take place in a vacuum and one key facet of the environment is the degree to which employees trust their employer. Brown *et al*. (2015) motivate the importance of trust within organizations using a principal–agent framework. With a risk averse agent and a risk neutral principal, resolution of the agency problem requires the principal to design a reward structure that will elicit high effort from the agent. But, if the agent has little or no experience of expending high effort, they will have little or no knowledge of the rewards and costs of taking this action, a problem exacerbated when the reward system is complex and there is volatility in the firm's environment, as in a recession. The authors show that trust can, to a degree, mitigate for this lack of information as it can elicit a performance enhancing effect and engender participation and co‐operation, or reduce costly resistance to productivity and/or quality enhancing change. In sum, trust can bring closer alignment between the goals of the principal and agent.

Using WERS data, Brown *et al*. (2015) find that employees' experiences during recession in terms of increased/re‐organized work, a wage freeze/cut, a reduction in non‐wage benefits, hours reduced, paid overtime restricted and a restriction on training all reduced employee trust. Only a requirement to take unpaid leave and a forced move to another job had no effect.

The trust measure (TRUST) comes from the combined European Values Survey/World Values Survey dataset and uses responses to the question: Generally speaking would you say that most people can be trusted or that you cannot be too careful in dealing with people? The surveys are conducted in waves and, on average, four observations are available for each country with linear interpolation/extrapolation used to construct a time series. There is temporal variation; between 2006 and 2012, Sweden's figure fell by over five percentage points. The most trusting population – over 70 per cent – was in Denmark and the least trusting was Cyprus with a figure of only 10 per cent.

## IV Empirical Strategy and Results

Empirically, the inter‐related nature of many of the dimensions of flexicurity is problematic due to high levels of correlation between the measures. To mitigate against this, Principal Components Analysis (PCA) was used to reduce the dimension of the data, mirroring ILO work (Auer and Chatani, 2010). To decide how many factors to keep, Glorfeld's method, which overcomes sample bias, was followed. Random data sets – 30 times the number of variables – were generated and PCA was conducted on each of these with the results being averaged. Only the two components with eigenvalues greater than one were kept. Looking first at F1, the highest factor loadings relate to ALMP, PLMP and LLL – security indicators – along with part‐time work. The second component, F2, is dominated by temporary work and self‐employment and, to a lesser extent, by unpaid family working, all measures of flexible forms of labour. With industrial democracy and trust added, they enter in the first factor. In both F1 + ID and F1 + ID+TRUST, industrial democracy (ID) carries the heaviest weighting in the PC whilst in F1 + TRUST the trust measure has the highest factor loading.

**Figure 1:**
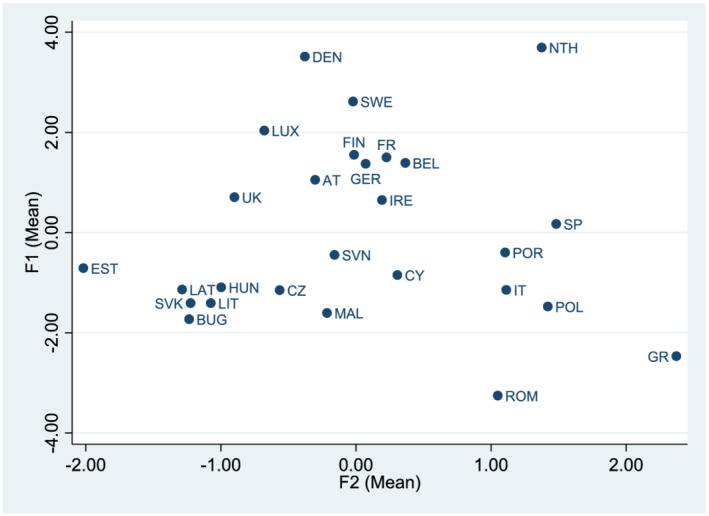
Flexicurity Indicators [Colour figure can be viewed at wileyonlinelibrary.com]

Figure 1 illustrates the relationship between the two baseline factors, F1 and F2. In terms of security, Denmark, the Netherlands and Sweden have the highest scores with the figure also showing the low levels of security offered to workers in the former CEE states. For flexibility, again the Netherlands scores highly, as do Greece, Italy, Poland, Portugal, Romania and Spain. With the exception of the Netherlands, all of these countries exhibit low security; only Spain has a score above the mean and it is only marginally so. In sum, it is clear that there is a good deal of heterogeneity among the sample countries and that flexibility alongside security is only strongly apparent in the Netherlands. 1 Also apparent is the fact that the variation in the security afforded to workers, which has a standard deviation of 1.78, exceeds the variation in flexibility with a standard variation of 1.05.

The second issue is endogeneity which necessitates the use of system GMM estimators. To overcome the problem of instrument proliferation, collapsed instrument sets were used and the number of instruments further reduced via PCA. 2 The empirical model is a variant of that used by Mankiw *et al*. (1992):

(1) $$∆\ln y_{\mathrm{it}}=\;a_{0,i}+\emptyset_{1}{\mathrm{lny}}_{i,t-1}+a_{1}\ln{\mathrm{INV}}_{i,t}+a_{2}\ln{\mathrm{HC}}_{i,t}+a_{3}{\text{lnPOPGRW}}_{i,t}\;\;+a_{4}∆\mathrm{DEF}+a_{5}2008+a_{6}F1+a_{7}F2+e_{i,t}$$

where ∆lny is the rate of growth of GDP per capita, INV is the share of investment in GDP, as a proxy for savings and HC is human capital, measured here as the percentage of the population with tertiary education. PGRW is a composite term $\left(n+g+d\right)$ where n is the population growth rate and following Islam (1995) and Mankiw *et al*. (1992), technical progress $\left(g\right)$ and the depreciation rate $\left(d\right)$ are assumed constant across countries and equal to 0.05. In order to examine the role of fiscal measures in the growth process, ∆DEF, the change in the government deficit measured relative to GDP, is in the empirical specification. Lagged GDP is included in the regressor set to capture convergence. The $a_{0,i}$ capture country heterogeneity, while e is the error term. A dummy is also included into the model to control for the 2008 crash. 3

Extensions of the empirical model involve the inclusion of industrial democracy:

(2) $$∆\ln y_{\mathrm{it}}=\;a_{0,i}+\emptyset_{1}{\mathrm{lny}}_{i,t-1}+a_{1}\ln{\mathrm{INV}}_{i,t}+a_{2}\ln{\mathrm{HC}}_{i,t}+a_{3}{\text{lnPOPGRW}}_{i,t}\;\;+a_{4}∆\mathrm{DEF}++a_{5}2008+a_{6}\left(F1+\mathrm{ID}\right)+a_{7}\left(F2+\mathrm{ID}\right)+e_{i,t}$$

and trust:

(3) $$∆\ln y_{\mathrm{it}}=\;a_{0,i}+\emptyset_{1}{\mathrm{lny}}_{i,t-1}+a_{1}\ln{\mathrm{INV}}_{i,t}+a_{2}\ln{\mathrm{HC}}_{i,t}+a_{3}{\text{lnPOPGRW}}_{i,t}+a_{4}∆\mathrm{DEF}\;\;+a_{5}2008+a_{6}\left(F1+\mathrm{ID}+\text{TRUST}\right)+a_{7}\left(F2+\mathrm{ID}+\text{TRUST}\right)+e_{i,t}.$$

Finally, considering trust without social partner engagement gives

(4) $$∆\ln y_{\mathrm{it}}=\;a_{0,i}+\emptyset_{1}{\mathrm{lny}}_{i,t-1}+a_{1}\ln{\mathrm{INV}}_{i,t}+a_{2}\ln{\mathrm{HC}}_{i,t}+a_{3}{\text{lnPOPGRW}}_{i,t}\;\;+a_{4}∆\mathrm{DEF}+a_{5}2008+a_{6}\left(F1+\text{TRUST}\right)+a_{7}\left(F2+\text{TRUST}\right)+e_{i,t}.$$

The results from the two‐step GMM estimations of the model are presented in Table 1. 4 All bar two parameter estimates are significant at the 5 per cent level, or better, and conform to expectations, insofar as investment and human capital promote growth, whereas the composite term incorporating population growth supresses it. The negative, significant, estimate for lagged GDP confirms the presence of conditional convergence. The positive coefficient estimate on the deficit variable, which is significant at the 1 per cent level, supports the growth dampening effect of austerity policies reported in Blanchard and Leigh (2014). The Hansen‐statistics for the model suggest that the instruments are appropriate and no second order autocorrelation is detected in the residuals.

**Table 1:** GMM‐Two‐step System Growth Regression Results, EU‐27§

	*Model 1*	*Model 2*		*Model 3*	*Model 4*
lnINV	0.2939*** (3.50)	0.2949*** (3.43)	lnINV	0.2871*** (3.10)	0.2816*** (2.93)
lnHC	0.1281** (2.06)	0.1376** (2.45)	lnHC	0.1305** (2.15)	0.1183* (1.77)
ln(n + g + d)	−0.5465** (2.18)	−0.5670** (2.23)	ln(n + g + d)	−0.5693** (2.03)	−0.5658** (2.02)
ΔDEF	0.0054*** (3.02)	0.0053*** (2.96)	ΔDEF	0.0055*** (3.07)	0.0055*** (3.03)
Error correction lny_−1_	−0.2253*** (3.21)	−0.2129*** (3.31)	Error correction lny_−1_	−0.2126*** (3.16)	−0.2250*** (3.10)
F1	0.0878*** (3.17)		F1 + ID+TRUST	0.0710*** (2.84)	
F2	0.0675** (2.13)		F2 + ID+TRUST	0.0656** (2.05)	
F1 + ID		0.0741*** (2.98)	F1 + TRUST		0.0839*** (3.17)
F2 + ID		0.0619** (2.27)	F2 + TRUST		0.0735* (1.89)
2008	−0.0090 (0.95)	−0.0072 (0.82)	2008	−0.0070 (0.85)	−0.0075 (0.84)
Constant	−0.6357 (0.69)	−0.8477 (0.97)	Constant	−0.8093 (0.90)	−0.6264 (0.68)
N	486	486	N	486	486
Instruments	26	26	Instruments	26	26
N PCs	6	6	N PCs	6	6
A‐B AR(1)	−1.94**	−1.91*	A‐B AR(1)	−1.90*	−1.91*
A‐B AR(2)	0.25	0.07	A‐B AR(2)	0.03	0.13
Hansen test	18.88	18.09	Hansen test	18.12	18.67
(p)	(0.40)	(0.45)	(p)	(0.45)	(0.41)

***, ** and * represent 1%, 5% and 10% significance levels respectively.

**Table 2:** Summary Statistic

	*Mean*	*Minimum*	*Maximum*	*Standard deviation*
GDP per capita growth	0.0357	−0.1771	0.3232	0.0471
Lagged GDP per capita Constant € at PPP	23959.47	4,900	77,600	11119.32
INV %GDP	22.2200	11.5000	37.4000	4.0196
TERT % population with tertiary education, ISCED 5,6,7 & 8	22.1090	2.8247	39.6000	7.9407
TEMP Temporary employees 15–64/total employees 15–64	0.0931	0.0060	0.2807	0.0555
SELF Self‐employed 15–64/total employees 15–64	0.1380	0.0614	0.3165	0.0503
PART Part‐time employees 15–64/total employees 15–64	0.1402	0.0087	0.4996	0.0963
UFW Unpaid family workers 15–64/total employees 15–64	0.0195	0.00004	0.1971	0.0283
LLL % of population 25–64	9.5912	0.8000	32.6000	7.2896
ALMP Active labour market policy expenditure	2545.11	2.1592	27227.3	2831.58
PLMP Passive labour market policy expenditure	3855.23	119.21	16789.5	3682.18
ID Industrial democracy index	52.97	36.73	70.94	10.76
TRUST % responding people could be trusted	31.77	2.74	86.86	17.61
ΔDEF Change in government deficit/GDP	0.91152	−18.2	18.5	2.4565

From Model 1, both of the standard flexicurity components serve to boost economic growth, with F1 – labour market policy and part time work – having the higher coefficient estimate and being slightly stronger statistically. With the inclusion of the social partners (Model 2), the results remain broadly similar, although the magnitude of both coefficient estimates fall. Including both industrial democracy and trust, as in Model 3, or trust alone, as in Model 4, both parameter estimates are significant, although only at the 10 per cent level for F2 + TRUST in Model 4.

Contextualizing the results for the flexicurity indicators is somewhat problematic insofar as they are artificial constructs. One approach to put some context on the interpretation of the results for the PCAs is to look at the coefficients on the indicators and the measured values for the sample countries. The coefficient on the security element (F1) is approximately 0.09 in Model 1 meaning that if F1 were to increase by one unit then this would stimulate growth by 0.09 per cent. As evident from Figure 1, for almost all western European nations – with the exception of Greece, Italy and Portugal – the maximum difference between their F1 value and that for the Netherlands which offers individuals the most labour market security, is 3.5 – in the case of Spain – so, if any of them matched the security position of this country, growth would be predicted to rise by between 0.02 per cent (Denmark) and 0.32 per cent (Spain). Using the alternative flexicurity indicators, including social partner engagement and/or trust produces similar findings.

As noted above, the standard deviation of the data is considerably smaller for the flexibility component. The difference between Greece with the most flexible labour market and Estonia with the least flexible one is approximately 4.39. With a coefficient estimate for F2 of 0.07, if Estonia pursued policies to bring its flexibility in line with that of Greece, then the potential growth dividend would be 0.39 per cent. Again, the findings are consistent when the augmented flexicurity PCs are used.

To provide an alternative interpretation of the results, a simulation exercise was undertaken wherein the flexibility and security values for the Netherlands were assigned to all sample countries. Overall, the average growth dividend resulting from this exercise was 0.34 percentage points for the security metric and 0.10 for flexibility. Romania made the highest gain from security with a figure of 0.62 percentage points. Denmark's gain was the smallest at only 0.02 percentage points, a finding that concurs with the fact that the country had the second highest value for security. Similarly, Greece with its highly flexible labour market, driven by self‐employment, would have seen its growth decline by 0.05 percentage points were its employment structure configured to that of the Netherlands whereas Estonia would have seen an increase of 0.39 percentage points.

For comparator purposes, simulation results were also performed for investment. Growth predictions were generated on the assumption that all countries had the same annual investment profile as the country with the highest figure, which was Estonia. Under this scenario, the average growth dividend would have been 0.07 but, for Greece, the figure for 2013 would have been 0.24. Annualizing these gains by country showed that across the whole sample period, the UK would have gained the most with an average increase in growth of 0.15 percentage points.

Although the differences in the coefficient magnitudes are relatively small, the growth dividend from the security component is greatest without the involvement of the social partners, and even in the absence of a trusting environment. This finding may reflect the fact that labour market policy is heavily loaded in this component and in the majority of countries – Sweden being a notable exception – social partner influence over such policy is low. Likewise, our trust measure does not refer specifically to the actors who determine the coverage of labour market actions. However, for the second flexicurity component, which is heavily dominated by temporary working and self‐employment, the coefficient estimate in Model 4 reflects the fact that workers feel more secure in atypical employment in trusting societies. 5

The results above all relate to the short‐run, instantaneous, coefficients. Setting $Y_{t}=Y_{t-1}$ gives the long‐run effects of the flexicurity PCs on per capita GDP. For F1 variants, the coefficients across the four models vary from 0.39 (Model 1) to 0.33 (Model 3). For F2 variants, the range is slightly lower, spanning 0.29 (Model 2) to 0.33 (Model 4). So, for the security component, the largest parameter estimate derives from the PC which excludes both industrial democracy and trust. The impact of the flexibility component shows slightly less variation with the two highest values coming from Models 3 and 4 which include trust in the PC, without or with, social partner engagement implying that trust and social partner can be important in economies characterised by a high penetration of temporary jobs and self‐employment.

## V ‘Flexicurity’ Countries during the Pandemic

In order to provide some insight as to how aspects of flexicurity affected economies during the pandemic, we look in some detail at five countries that ranked highly on the flexibility and/or security criteria. Those selected are Denmark (security), the Netherlands (flexibility and security), Poland (flexibility), Spain (flexibility and security – social security payments) and Sweden (security). The countries were evaluated at the end of the Great Recession in 2009 with a robustness check confirming the consistency of the results for 2010. In terms of relevant policy changes between the two recessions, in Spain strict social policy reforms came as a condition of the country's bailout package eroding the country's social security pillar with a lower base being brought in for contributory payments and restricted benefits for older workers. In Denmark, Sweden and the Netherlands, conditions for agency and other temporary workers were improved, although Denmark and the Netherlands increased conditionality and a more workfare approach was applied to both ALMP and LLL. Changes in Poland, a country with low spending on labour market support, primarily concerned enabling parents to return to work.

During the pandemic, countries eschewed promoting job‐to‐job movements in favour of supporting workers in their current employment. In fact, Denmark, the Netherlands, Spain and Sweden already had permanent job retention schemes in place; see Drahokoupil and Müller (2021). Poland also adopted a short‐time working scheme as did the Netherlands which used this measure to replace its permanent furlough scheme. Of course, the generosity of the schemes varied considerably with Denmark and Netherlands affording their workers 100 per cent of their wage whilst Poland only offered 50 per cent. Top spenders were the Netherlands and Spain with figures of 1.9 per cent and 1.8 per cent of GDP respectively, whilst Poland only spent 0.3 per cent of GDP. At the peak in 2020, almost 35 per cent of all employees in the Netherlands were on a JRS scheme, along with almost 20 per cent in Spain. In Poland, the figure was below 5 per cent.

In terms of social partner involvement, trade unions and other company‐level employee representatives have played a role in the design and implementation of job retention schemes. In Denmark there was a formal requirement that the social partners be involved in negotiations surrounding the permanent furlough scheme, but when the COVID‐19 scheme was introduced, this requirement was loosened so that the social partners were only informed or consulted. For wage subsidies, involvement in negotiations was required in Poland but only consultation was necessary in the Netherlands. Likewise, in both Denmark and Sweden, social partners helped negotiate short‐time working agreements but were only consulted/informed in Spain. In general, the role for worker participation was greater in countries where permanent job retention schemes were in place than they were in countries where they were newly established.

The degree to which these schemes shielded the labour market is reflected in the employment figures which show that over the period 2019Q1 to 2020Q2 employment only contracted by some 3.4 per cent across the Union and, by Q2 2021, employment levels had exceeded the earlier figure. Four of the countries considered here fared better, although Spain recorded employment losses of almost 8 per cent. Overall though, unemployment did not increase to the extent that had been predicted early on in the pandemic. However, these figures conceal the fortunes of the flexible workforce. The Netherlands, Poland, Spain and Sweden all feature in the top‐5 EU‐27 countries for temporary jobs, ranging from 21 per cent in Poland and Spain to 15.6 per cent in the Netherlands. Between 2019Q4 and 2020Q2, some 15.5 per cent of temporary positions were lost across Europe. In Spain the figure exceeded 21 per cent.

All five countries suffered a significant fall in GDP in the second quarter of 2020 with Spain suffering the most with a drop in GDP of almost 18 per cent, although this country also enjoyed the largest rebound. Over the period 2019Q4 to 2021Q3, Spain's GDP fell 0.4 per cent with all other countries managing positive growth; Denmark (0.34 per cent), the Netherlands (0.36 per cent), Poland (0.54 per cent) and Sweden (0.38 per cent), all exceeding the EU average of 0.16 per cent. Overall, these figures indicate a bounce back that was stronger than that for the Great Recession.

The Economist (2021), rated 23 countries across five measures – GDP, household income, investment, public debt and share prices – ranking Denmark, the Netherlands, Poland, Sweden and Spain 1st, 8th, 6th, 3rd and last, respectively, highlighting Spain as the weakest performer. This country suffered large‐scale infections and strict lockdowns but the structural features of its economy made it vulnerable. 12 per cent of the economy is dependent on contact‐intensive service sectors, notably tourism and hospitality. Workers here tend to be young, low‐skilled and frequently on temporary contacts. These workers were similarly affected in the Netherlands, but the country is far less dependent on contact‐intensive sectors (IMF, 2021). Spain also entered the pandemic with weak macroeconomic fundamentals; very high public debt and unemployment. Denmark's performance has been attributed by some to be at least partially attributable to trust (Olagnier and Mogensen, 2021) with the authors noting that Danish citizens immediately followed all the rules as they inherently trust politicians. 6 They also note that Danes always respect social distancing and have far fewer contacts than many other European nationals.

Sweden entered the pandemic with strong public finances with low productivity businesses having relocated to low cost economies and Sweden's industrial companies focusing on knowledge‐intensive activities such as research and development, design and logistics. Poland's relatively strong performance during the pandemic has been attributed by some to be down to ‘luck’– that is, factors beyond the direct control of government policies (Bukowski and Paczos, 2021). Not only is the country ‘semi‐peripheral’, meaning that the virus arrived relatively late giving the government time to prepare, 40 per cent of the population live in low‐density rural areas. In addition, the country is large and its economic structure, less service sector dependent, made it more resilient.

## Summary and Concluding Remarks

Early evidence suggested that major economies weathered the pandemic reasonably well. Output recovered relatively quickly and job losses never came near the levels suggested due to the unprecedented fiscal stimulus that advanced financial support to keep people in jobs. However, as countries emerge from the pandemic they do so with a legacy of high levels of debt. At the end of 2021, 14 Member States had a debt‐to‐GDP ratio that exceeded the 60 per cent Maastricht limit, with the figures for Greece and Italy standing at 193 per cent and 151 per cent, respectively. Looking back to the financial crisis, after an initial fiscal policy stimulus, the struggling European countries (Spain, Greece and Portugal) switched dramatically to austerity to bring debt levels under control but GDP fell so much that debt to GDP ratios actually rose. It is now accepted that following the financial crisis the eurozone adopted the wrong policy mix as insufficient co‐ordination between countries led to a premature withdrawal of fiscal support. This, coupled with sluggish structural reforms, caused the eurozone to return to recession.

Although the virus is now receding across Europe, rising energy prices, the war with Ukraine and ongoing supply shortages once again bring the threat of recession and governments may look to productivity increases to overcome this. Offering innovation‐led growth flexicurity policies might potentially aid this agenda. There are, however, observers such as Hastings and Heyes (2016) and Heyes (2011, 2013) who contend that, since the financial crisis, countries have placed less weight on security leading to a de facto demise of flexicurity. That said, the EU's Recovery and Resilience Plan carries strong elements of both security and flexibility. More than one‐third of the proposals involve labour market activation focusing mainly on the low skilled, the young and the long‐term unemployed (Schulz, 2020). There is also an emphasis on the necessity for adequate social assistance. Furthermore, the ECB (2020) recommends boosting human capital through on‐the‐job training and active labour market policies, both of which are components of flexicurity. Overall, the focus is not on protecting workers from the market but, instead, the aim is to adapt workers' skills to what the market needs.

The results in this paper provide some support for the role of flexicurity policies in providing a positive impetus to growth. From the results presented, it was the security elements – lifelong learning, labour market activation policies and labour market supports – that exerted the largest positive growth impetus and this finding remained robust when measures for industrial democracy and trust were included in the model, although these additional components did moderately dampen the effect. The flexible work elements comprising primarily temporary work and self‐employment also boosted growth, albeit by a lower order of magnitude. For flexibility, the growth effect was maximized when trust was included in the PC specification. Using simulations to overcome the fact that principal components are artificial constructs rendering interpretation problematic, it was demonstrated that countries could boost performance if they adopted a flexicurity policy agenda.

## Supporting information


**Table S1.** Flexicurity in the EU 2000–17.

## Footnotes

^1^
Including ID and TRUST into the PCs produces a very similar pattern.

^2^
Levin‐Lin‐Chu tests rejected the null of unit roots for the data.

^3^
Aside from the flexicurity measures, the model differs from the Mankiw *et al*. (1992) framework which uses a level (the neo‐classical) approach as opposed to a growth rate (the new growth theory approach) as the dependent variable.

^4^
Summary statistics are provided in Table 2.

^5^
Data limitations preclude running the model on sub‐groups of countries but the model was estimated excluding one country at a time as a robustness check.

^6^
A recent study found that 95 per cent of Danes felt that their government had handled the pandemic well, for Spain the figure was only 54 per cent (Pew Research Center, 2020).
