# Supplementary material for: COVID‐19, the Great Recession and Economic Recovery: A Tale of Two Crises
Source: J Common Mark Stud. 2022 Jul 7:10.1111/jcms.13383. Online ahead of print. doi: 10.1111/jcms.13383 (PMC9349527; doi:10.1111/jcms.13383)
Supplement: Supplementary file 1 — Table S1. Flexicurity in the EU 2000–17. [file JCMS-9999-0-s001.docx]

Appendix Table 1: Flexicurity in the EU 2000-2017†

| Country | PART | TEMP | SELF | UFW | LLL | ALMP | PLMP | TRUST | ID |
| --- | --- | --- | --- | --- | --- | --- | --- | --- | --- |
| Austria  Belgium  Bulgaria  Cyprus  Czech Rep.  Denmark  Estonia  Finland  France  Germany  Greece  Hungary  Ireland  Italy  Latvia  Lithuania  Luxembourg  Malta  Netherlands  Poland  Portugal  Romania  Slovakia  Slovenia  Spain  Sweden  UK | 22.53  22.05  2.39  8.84  4.88  23.22  8.00  13.32  17.37  24.02  6.36  4.60  19.09  13.69  7.91  8.27  16.33  10.52  46.41  8.11  9.09  9.72  3.40  8.19  11.90  23.55  24.84 | 7.66  7.40  4.78  11.44  6.88  8.71  2.70  14.15  13.22  12.03  7.58  7.36  6.45  9.31  5.35  3.32  5.77  4.59  14.81  17.53  17.01  1.19  5.23  13.76  23.62  14.31  5.15 | 10.94  13.23  12.11  16.64  15.86  7.98  8.35  12.14  10.31  10.06  29.86  12.02  15.16  22.82  10.07  12.60  7.73  13.32  12.64  19.40  18.42  18.68  12.77  10.84  16.48  9.40  12.73 | 1.47  1.28  1.21  1.73  0.01  0.01  0.30  0.38  0.73  0.61  6.05  0.44  0.79  2.17  2.05  2.12  0.45  6.23  0.55  3.84  1.04  12.70  0.11  3.60  1.13  0.21  0.26 | 12.34  7.22  1.68  6.77  7.71  27.27  9.58  22.61  8.54  7.30  2.62  3.85  6.61  6.14  6.78  4.93  10.98  6.06  16.91  4.45  6.35  1.37  3.37  12.83  8.74  23.33  20.25 | 3037.86  4005.20  325.25  480.98  1175.31  8487.18  376.72  3446.52  4491.61  4271.84  2993.85  1413.67  4080.34  1290.95  247.12  563.23  6948.82  644.61  6713.14  730.70  1820.69  168.97  471.22  929.34  1459.58  6598.02  1537.44 | 5951.14  11184.08  416.38  3360.47  947.67  9200.70  739.55  5972.58  8923.06  6339.27  1443.20  872.62  7158.25  2440.21  466.30  483.51  10089.11  1170.57  9645.64  779.63  4065.00  413.26  846.88  1616.65  4175.86  4734.79  834.79 | 36.86  34.16  18.43  9.85  30.34  76.25  35.71  64.62  18.11  39.19  21.34  22.21  38.94  30.03  26.26  31.29  31.40  21.71  59.27  22.59  17.56  11.54  12.73  19.69  23.03  63.64  42.06 | 67.42  64.49  37.58  47.74  49.09  70.86  47.69  67.52  58.11  63.53  43.77  44.14  55.67  48.82  39.50  41.24  67.98  49.29  68.47  39.52  48.62  36.80  43.89  56.63  52.01  68.63  51.24 |

† Data from Eurostat (<http://ec.europa.eu/eurostat/data/database>). ALMP and PLMP from <https://webgate.ec.europa.eu/empl/redisstat/databrowser/explore/all/lmp?display=card&sort=category> supplemented from OECD ([http://stats.oecd.org](http://stats.oecd.org/)). TRUST from the European Values Survey ([www.europeanvaluesstudy.eu](http://www.europeanvaluesstudy.eu/)) and the World Values Survey ([www.worldvaluessurvey.org](http://www.worldvaluessurvey.org/)). ID from <https://www.eurofound.europa.eu/data/Industrial-relations-index?period=2013-2017&mode=all&country=all&breakdown=index>.
